# Effects of Incretin-Based Therapies on Neuro-Cardiovascular Dynamic Changes Induced by High Fat Diet in Rats

**DOI:** 10.1371/journal.pone.0148402

**Published:** 2016-02-01

**Authors:** Silvio Rodrigues Marques-Neto, Raquel Carvalho Castiglione, Aiza Pontes, Dahienne Ferreira Oliveira, Emanuelle Baptista Ferraz, José Hamilton Matheus Nascimento, Eliete Bouskela

**Affiliations:** 1 Laboratory for Clinical and Experimental Research on Vascular Biology (BioVasc), Biomedical Center, State University of Rio de Janeiro, Rio de Janeiro, RJ, Brazil; 2 Cardiac Electrophysiology Laboratory, Carlos Chagas Filho Institute of Biophysics, Federal University of Rio de Janeiro, Rio de Janeiro, RJ, Brazil; Hosptial Infantil Universitario Niño Jesús, CIBEROBN, SPAIN

## Abstract

**Background and Aims:**

Obesity promotes cardiac and cerebral microcirculatory dysfunction that could be improved by incretin-based therapies. However, the effects of this class of compounds on neuro-cardiovascular system damage induced by high fat diet remain unclear. The aim of this study was to investigate the effects of incretin-based therapies on neuro-cardiovascular dysfunction induced by high fat diet in Wistar rats.

**Methods and Results:**

We have evaluated fasting glucose levels and insulin resistance, heart rate variability quantified on time and frequency domains, cerebral microcirculation by intravital microscopy, mean arterial blood pressure, ventricular function and mitochondrial swelling. High fat diet worsened biometric and metabolic parameters and promoted deleterious effects on autonomic, myocardial and haemodynamic parameters, decreased capillary diameters and increased functional capillary density in the brain. Biometric and metabolic parameters were better improved by glucagon like peptide-1 (GLP-1) compared with dipeptdyl peptidase-4 (DPP-4) inhibitor. On the other hand, both GLP-1 agonist and DPP-4 inhibitor reversed the deleterious effects of high fat diet on autonomic, myocardial, haemodynamic and cerebral microvascular parameters. GLP-1 agonist and DPP-4 inhibitor therapy also increased mitochondrial permeability transition pore resistance in brain and heart tissues of rats subjected to high fat diet.

**Conclusion:**

Incretin-based therapies improve deleterious cardiovascular effects induced by high fat diet and may have important contributions on the interplay between neuro-cardiovascular dynamic controls through mitochondrial dysfunction associated to metabolic disorders.

## Introduction

Obesity is a worldwide chronic disease characterized by excess of body fat, resulting from an imbalance in energy metabolism influenced by behavioral, environmental, cultural and genetic factors [1]. Recent statistics from the World Health Organization (WHO) reported that the prevalence of overweight (body mass index between 25 and 30 kg m^-2^) exceeded 1.4 billion adults, and 200 million men and 300 million women were considered obese (body mass index above 30 kg m^-2^) [2]. Therefore, obesity is a serious public health problem and leads to an increase in the prevalence of dyslipidemia, insulin resistance (IR), type 2 diabetes mellitus (T2DM) and cardiovascular diseases (CVDs) [2]. In this context, CVDs is the leading cause of death worldwide, with an estimation of 17.3 million deaths per year, being 7.3 million from coronary heart disease (CHD) and 6.2 million from stroke. The number of deaths from CVDs, mainly CHD and stroke, should increase to reach 23.3 million by 2030. Strategies to reduce this projection have to take into consideration the prevention of the disease and the need to address its risk factors, mainly unhealthy diet and obesity [3].

Regarding obesity control, at present bariatric surgery is the only successful treatment for severe obesity. However, it is not the best option due to its costs, shortage of bariatric surgeons, side effects and post-surgery complications. In this context, pharmacological strategies should also be considered for obesity control [4]. One possibility could be the administration of glucagon-like peptide-1 receptor (GLP-1R) agonists or dipeptidyl peptidase-4 (DPP-4) inhibitors, two incretin-based therapies commonly used for the treatment of type 2 diabetes mellitus due to their effects on body weight reduction. It should be noted that the effectiveness of incretin-based therapies in reducing the deleterious effects of high fat diet on the cardiovascular system is not well elucidated [4–5].

For this reason, our aim was to investigate incretin-based therapies with GLP-1R agonist and DPP-4 inhibitor in a perspective of integrative physiology based on metabolic and neuro-cardiovascular dynamic control. Our results show that incretin-based therapies could act on deleterious cardiovascular effects induced by high fat feeding and may have important contributions on the interplay between neuro-cardiovascular dynamic controls.

## Materials and Methods

### Animal care

The study followed the Principles of Laboratory Animal Care published by US National Institute of Health (NIH publication, 1985) and was approved by the State University of Rio de Janeiro Committee for Animal Experimentation. During invasive procedures the animals were sbmitted to ketamine/xylazine anesthesia (90/10 mg kg-1, i.p.) and animals were monitored by the researchers until they were awake and well. All efforts were made to minimize suffering.

Male Wistar rats (n = 24) were used in the experiments and immediately after the weaning period divided into two initial groups, high fat diet (n = 16) and lean control group (CTRL, n = 8) (Table 1). Eighteen weeks, after the start of high fat diet, rats were subdivided into three subgroups: (1) high fat diet (HFD, n = 6), (2) high fat diet treated with GLP-1R agonist Liraglutide (HFD Liraglutide, n = 5) and high fat treated with DPP-4 inhibitor Sitagliptin (HFD Sitagliptin, n = 5). During 4 weeks, HFD Liraglutide and HFD Sitagliptin subgroups were treated with Liraglutide (0.6 mg kg^-1^, s.c once a day) and Sitagliptin (4 mg kg^-1^, in the drinking water), respectively. Body weight and food intake were measured weekly and the animals were housed in a temperature-controlled room (23±2°C) on a 12:12 h dark/light cycle with free access to rat chow and water. Physical conditions of the animals were monitored four times a week.

**Table 1:** Composition of standard chow and high-fat diet.

Ingredient	Standard chow		High fat diet	
	g kg^-1^	kcal kg^-1^	g kg^-1^	kcal kg^-1^
Cornstarch (Q.S.P)	530	2120	294	1178
Casein	220	880	220	880
Ether Extract	40	360	40	360
Lard	-----	-----	235	2115
Soybean Oil	70	630	70	630
Cellulose	80	-----	80	-----
Mineral mix	35	-----	35	-----
Vitamin mix	10	-----	10	-----
L-Cystine	3	-----	3	-----
Choline	2.5	-----	2.5	-----
Total	1000	3990	1000	5163

### Fasting glucose and insulin tests

At the end of the experimental period, rats were subjected to a 6-hour fasting and glucose and insulin tolerance (ITT) tests were performed. Fasting glucose was quantified using the glucose meter (OneTouch^®^ Ultra^®^, Milpitas CA, USA). Five minutes after the first glucose collection, 1.5 IU kg^−1^ of human recombinant insulin (Novolin^®^, Novo Nordisk A/S, Kalundborg, Denmark) was injected i.p. and then blood samples from the tail were collected at 0, 5, 10, 15, 20, 25 and 30 min for serum glucose determination. The rate constant for plasma glucose disappearance (k_ITT_) was calculated using the formula, 0.693/biological half-life (*t*_1/2_) and plasma glucose *t*_1/2_ from the slope of the least squares analysis of its plasma concentration during the linear phase of decline [6].

### Analysis of heart rate variability

Subcutaneous electrodes were implanted at least 48 h before ECG recordings under anaesthesia (ketamine/xylazine: 90/10 mg kg^-1^, i.p.). The electrodes consisted of two steel wires (0.4 mm in diameter) positioned in the torso (the upper right and lower left quadrants) and electrocardiograms (ECG) were recorded (emulating Lead II) from conscious rats, for 5 min. Signals were acquired with a sampling rate of 10 kHz and resolution of 16 bits (PowerLab 4/35 and software LabChart 7.0, AD Instruments, Colorado, CO, USA). The amplifier (differential A/C amplifier model FE 136, Animal Bio Amp, AD Instruments, Colorado, CO, USA) cables were connected to electrodes of unrestricted animals that had been resting quietly in individual cages for at least 20 min prior to recording to minimize stress. In order to reduce potential effects of circadian fluctuations, all recordings were conducted at the same time of the day (13:00–15:00 h).

Heart rate variability (HRV) analysis was performed as previously described [7]. The following indexes were obtained in the time domain: mean R–R interval (R–R), square root of the mean squared differences of successive R–R intervals (RMSSD) and percentage of successive R–R interval differences greater than 5 ms (pNN5). For spectral (frequency domain) analysis of HRV, both low-frequency (LF: 0.2–0.8 Hz) and high-frequency (HF: 0.8–2.5 Hz) bands were determined and the LF/HF ratio was used as an index of sympathovagal balance.

### Cerebral Microcirculation

Initially, rats were anesthetized (ketamine/xylazine: 90/10 mg kg^-1^, i.p.) and placed in a stereotaxic frame for head fixation to reduce animal movement during image acquisition (AVS Projetos, São Carlos, SP, Brazil). An incision of 4 to 5 cm down the midline of the scalp was made using a scalpel blade. The scalp was retracted to the side of the head with four hemostats, two placed on either side caudally and two rostrally. Bleeding was controlled with soaked gauze with modified artificial cerebral spinal fluid (mACSF). A relatively large cranial window (4 × 6 mm^2^) was generated through an electric powered drill (Beltec, LB-100, Araraquara, SP, Brazil), with the bone completely removed and the dura mater carefully resected for optical access. After the surgical procedure, the cortical surface was continuously flushed with mACSF and small pieces of SurgiFoam presoaked in mACSF were applied to control additional bleeding. Rapidly, the window was sealed with 1.5% (w/v) low-melting point agarose dissolved in mACSF to avoid hemorrhage, brain swelling and imaging quality degradation. After the bone was removed, a PE-10 catheter was inserted into the femoral vein to deliver 0.4 mL volume of 5% fluorescein-dextran (FITC-dextran 150) dissolved in saline to label blood serum (2MDa, Sigma Aldrich, FD2000S). When necessary, Ringer’s lactate solution was injected intraperitoneally at a volume of 3 mL per kg every 2 hours to maintain body fluids and energy requirements [8].

Sequentially, cortical intravital microscopy (AxioScopeA1, Carl Zeiss, Oberkochen, Germany) was carried out and images of cerebral microcirculation were acquired using an AxioVision software 4.7 (Carl Zeiss, Oberkochen, Germany). Capillary diameters were measured and the functional density (number of capillaries with flowing red blood cell by unity tissue area) determined using the AxioVision software 4.7 (Carl Zeiss, Oberkochen, Germany). Serial images were taken with ×10 ocular, ×10 objective/0.25 lenses (Axiocam HR; Carl Zeiss, Oberkochen, Germany). Only perfused capillaries were counted to determine the mean functional capillary density, expressed as number of capillaries mm^-2^.

### Arterial blood pressure and left ventricular function

Rats were anesthetized (ketamine/xylazine: 90/10 mg kg^-1^, i.p.) and a polyethylene catheter (PE-50) filled with heparinized saline was inserted into the right carotid artery to record systolic (SBP) and diastolic (DBP) arterial blood pressures. After arterial blood pressure measurements, the catheter was advanced into the left ventricle (LV) to measure systolic and diastolic pressures and their first time derivatives (positive and negative, maximum dP/dt^+^ and minimum dP/dt^-^, respectively).

All arterial and ventricular measurements were registered in real-time and continuously recorded in a four-channel data acquisition system (PowerLab 4/35, AD Instruments, Colorado, CO, USA). Signals were analyzed off-line using LabChart software (version 7.0, AD Instruments, Colorado Springs, CO, USA). All data were registered for ≥5 min. After the treatment period, rats were euthanized with an anesthetic overdose followed by exsanguination and their tissues (when necessary) weighted and designed to mitochondrial swelling assay.

### Mitochondrial isolation and swelling protocol

Mitochondria from rat brains and hearts were isolated as previously described [9]. These tissues were homogenized in isolation buffer (250 mM Sucrose, 1 mM EGTA, 5 mM HEPES, pH 7.3) containing 1 mg of subtilisin A (Sigma, St. Louis, MO, USA) per g of tissue and centrifuged at 1,000 × g for 10 min at 4°C. The pellet was discarded and the supernatant centrifuged again at 12,000 × g for 15 min. Briefly, 1 mg ml^-1^ of heart mitochondria was suspended in mPTP buffer (200 mM Sucrose, 10 mM HEPES, 5 mM KH_2_PO_4_, 10 μM EGTA, pH 7.3) supplemented with 10 mM succinate and 1.5 mM rotenone. Mitochondrial swelling was triggered by the addition of 1 mM of CaCl_2_ to brain and heart tissues and monitored by the relative decrease in percentage of light-scattering at 540 nm in a Spectramax M2 spectrophotometer (Molecular Devices, CA, USA) for 21 minutes.

### Statistical analyses

Data are presented as mean ± SEM, unless otherwise noted. Multiple comparisons were performed by one-way ANOVA followed by Newman-Keuls post-hoc test. The two-way repeated-measures ANOVA was used to analyze changes in mitochondrial swelling over time. *p* <0.05 was considered to indicate statistical significance.

## Results

### Biometric, cumulative caloric intake and metabolic results

In order to investigate the role of incretin-based therapies in the attenuation of deleterious effects on metabolic, cardiovascular and neurovascular functions induced by high fat diet, we have treated for 4 weeks two distinct subgroups of rats with liraglutide and sitagliptin previously fed with high fat diet during 18 weeks and assessed metabolic, cardiovascular, neurovascular and mitochondrial parameters.

Table 2 illustrates biometric results, cumulative caloric intake during the treatment period and blood glucose levels of the experimental groups. After 18 weeks of high fat diet, HFD rats showed an increase of approximately 18% in body weight compared with control group. Four weeks of liraglutide treatment normalized body weight of high fat animals, reducing it in 25% in relation to HFD rats. However, sitagliptin treatment showed similar values to HFD rats (n.s) and significantly greater values than CTRL (approximately 11%) and HFD Liraglutide (approximately 25%).

**Table 2:** Biometric, cumulative caloric intake, glucose and myocardial parameters after diets and incretin-based therapies.

Variable	Groups			
	CTRL	HFD	HFD Liraglutide	HFD Sitagliptin
Biometric results				
BW (g)	397.7±8.1	467.8±18.4*	351.6±8.8^†††^	440.3±23.9*/^‡‡^
EF (g)	5.52±0.94	11.21±1.50*	5.49±0.80^†^	11.20±2.15^‡^
RF (g)	5.58±1.85	15.93±1.62**	6.11±0.25^†^	20.71±3.15^‡‡‡^
VF (g)	3.82±0.17	11.69±1.23**	3.31±0.43^††^	11.15±3.26^‡‡^
Cumulative caloric intake (kcal)	3024±66.74	2814±32.10	2899±56.46	2941±57.39
Fasting glucose (mmol L^-1^)	4.9±0.3	6.6±0.7***	5.1±0.3^††^	6.4±0.6^‡‡^
ITT parameters				
K_ITT_ (% min^-1^)	4.1±0.7	1.8±0.2*	4.5±0.5^††^	2.1±0.3^‡^
AUC	2180±197	3570±181***	2634±158^††^	2905±132
Myocardial parameters				
HW (g)	1.15±0.03	1.54±0.05*	1.02±0.05^†††^	1.18±0.06^†††^
BW/HW (g kg^-1^)	2.74±0.12	3.39±0.18*	2.49±0.18^†^	2.42±0.14^†^

BW, body weight; EF, epydidimal fat; HW, heart weight; RF, retroperitoneal fat; VF, visceral fat.

*p<0.05 vs. CTRL;

**p<0.01 vs. CTRL;

*** *p<*0.001 vs. CTRL;

^**†**^*p<*0.05 vs. HFD;

^**††**^*p<*0.01 vs. HFD;

^**†††**^*p<*0.001 vs. HFD;

^‡^*p<*0.05 vs. CTRL and HFD Liraglutide;

^‡‡^*p<*0.01 vs. CTRL and HFD Liraglutide

^‡‡‡^*p<*0.001 vs. CTRL and HFD Liraglutide. Data are shown as the mean ± SEM; *n* = 5–8.

To further investigate whether incretins could decrease body weight through fat reduction, we have evaluated fat weight depots in all experimental groups. After 18 weeks, HFD presented bigger epididymal, retroperitoneal and visceral fat depots compared with CTRL rats, while HFD Liraglutide ones showed fat depots with weight similar to CTRL rats.

Although sitagliptin is largely used as an effective DPP4 inhibitor to prolong endogenous action of GLP-1, the HFD Sitagliptin subgroup did not show reduction of fat depots and presented values similar to HFD rats.

To evaluate if glycaemic disorders associated to an increased adiposity could be influenced by incretin therapies, we have assessed fasting and glucose time course through ITT. As expected, higher fasting glucose and insulin resistance were observed in HFD rats compared with CTRL ones. In parallel, HFD Liraglutide subgroup presented results similar to CTRL and significantly different from HFD. No significant differences in fasting glycaemia and ITT parameters were found between HFD and HFD Sitagliptin rats, suggesting that glycaemic disorders associated to high adiposity were attenuated only by liraglutide therapy.

To further investigate whether heart weight and cardiac hypertrophy could be attenuated with incretin-based therapies, we have evaluated both of them in the experimental groups. These indexes were increased in HFD rats compared with CTRL ones, while HFD Liraglutide reduced these parameters to CTRL values. In addition, although sitagliptin treatment did not affect biometric parameters, glucose levels and insulin resistance in rats fed with high fat diet, heart weight and cardiac hypertrophy indexes were reduced (Table 2).

No differences were observed between CTRL and control animals after liraglutide and sitagliptin treatments on biometric, cumulative caloric intake, glucose and myocardial parameters (data not shown).

### Haemodynamic and left ventricular parameters

**Fig 1:**
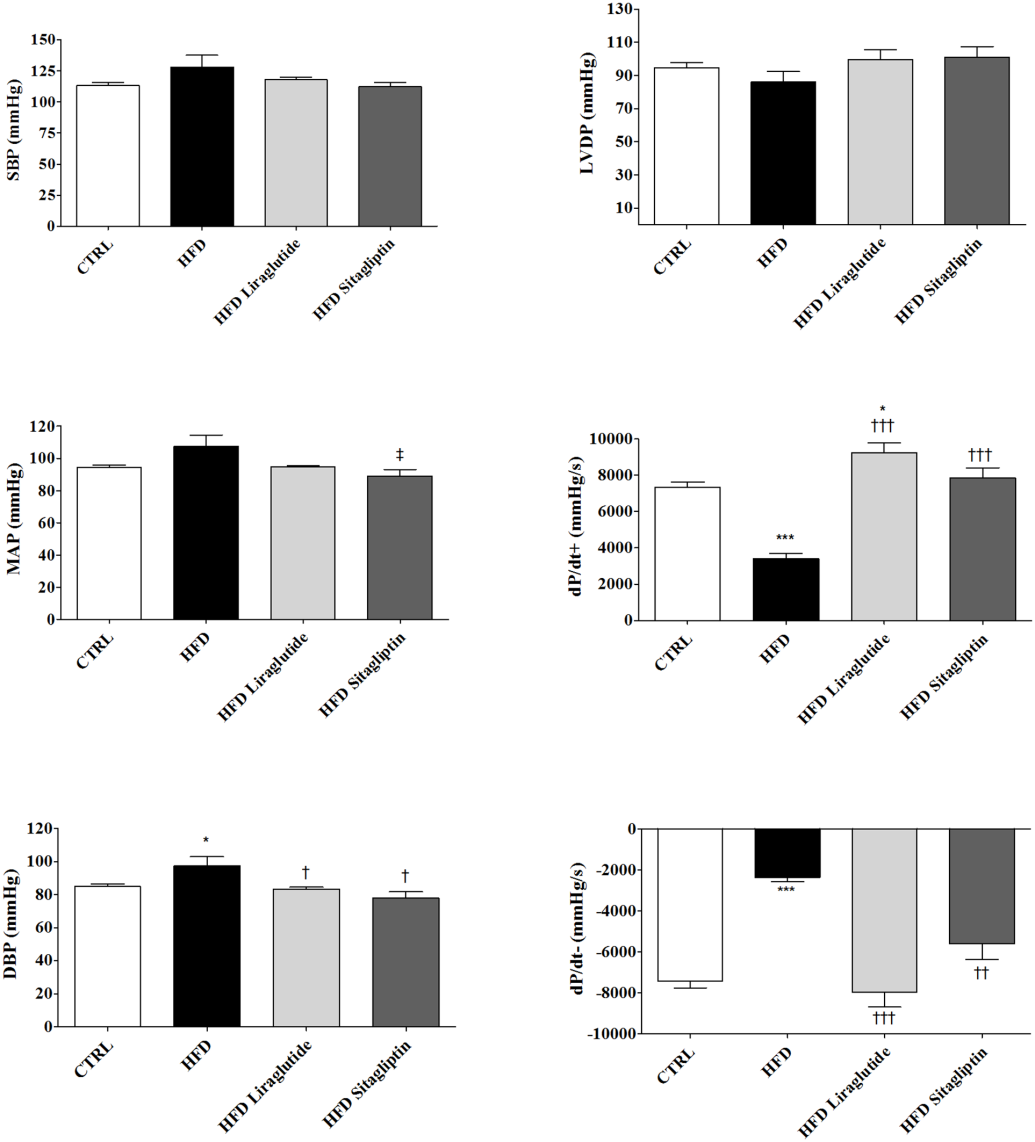
Haemodynamic and LV parameters after HFD and incretin-based therapies. Data are shown as mean ± SEM; *n* = 5–8. **p<0.01 vs. CTRL; ***p<0.001 vs. CTRL; ^**†**^*p<*0.05 vs. HFD; ^**††**^*p<*0.01 vs. HFD and ^**†††**^*p<*0.001 vs. HFD.

To investigate the influence of incretin-based therapies in attenuation of deleterious effects on cardiovascular parameters induced by high fat diet, we have assessed systemic (MAP) and left ventricular pressures at the end of the experimental period. Although systolic blood pressure did not differ between groups, only HFD Sitagliptin rats presented reduced MAP response and HFD group showed increased DBP compared with CTRL rats, while liraglutide and sitagliptin normalized DBP compared to HFD group. Although LVDP presented similar results in all groups, HFD rats showed marked reduction of contractility (dP/dt^+^) and relaxation (dP/dt^-^) indexes compared with CTRL ones. In parallel, HFD Liraglutide group presented higher dP/dt^+^ and dP/dt^-^ indexes compared with HFD and CTRL subgroups, while HFD Sitagliptin subgroup showed increased dP/dt^+^ and dP/dt^-^ indexes compared with HFD, but not to CTRL group, suggesting that cardiovascular disorders associated with high fat diet were reversed with both incretin therapies (Fig 1).

No differences were observed between CTRL, CTRL Liraglutide and CTRL Sitagliptin regarding SBP, LVDP, MAP, DBP and dP/dt^+^ parameters. However, CTRL Liraglutide group has a lower relaxation index (dP/dt^-^) than CTRL group (data not shown).

### Analyses of heart rate variability

**Fig 2:**
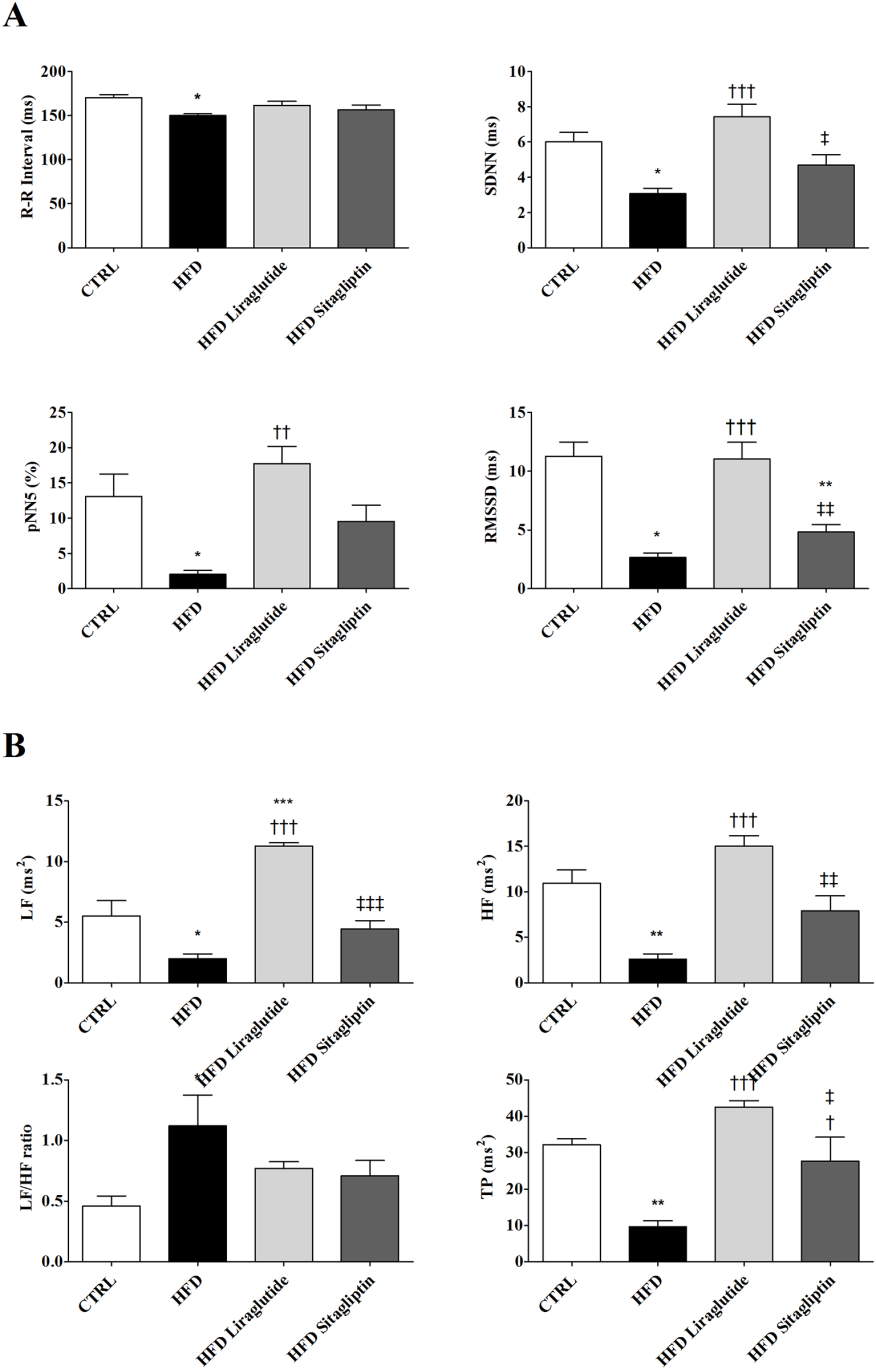
Effects of high fat diet and incretin-based-therapies on autonomic nervous system. **(a)** Effects of high fat diet alone or in combination with liraglitude or sitagliptin treatments on R-R intervals and time-domain parasympathetic indexes of heart rate variability (SDNN, pNN5% and RMSSD). (**b**) Effects of high fat diet alone or in combination with liraglitude or sitagliptin treatments on frequency-domain of heart rate variability (SDNN, pNN5% and RMSSD). High-frequency (HF), low- frequency (LF) power spectra and the ratio between low-frequency to high-frequency (LF HF^-1^ ratio) power spectra are shown. The following groups (n = 5 each) were examined: Control (CTRL), high fat diet (HFD), high fat diet plus liraglutide (HFD Liraglutide) and high fat diet plus sitagliptin (HFD Sitagliptin). Data are shown as mean ± SEM; *n* = 5–8. *p<0.05 vs. CTRL; **p<0.01 vs. CTRL; ^†^*p<*0.05 vs. HFD; ^†††^*p<*0.001 vs. HFD; ^‡^*p<* 0.05 vs. HFD Liraglutide; ^‡‡^*p<*0.01 vs. HFD Liraglutide and ^‡‡‡^*p<*0.001 vs. HFD Liraglutide.

In order to analyze the effects of high fat diet and incretin-based-therapies on autonomic nervous system, time and frequency-domains indexes of heart rate variability (HRV) were assessed using Matlab-based algorithms. HFD significantly reduced R-R intervals compared with the control group and no significant effects of both treatments could be observed. Time-domain indexes of HRV (SDNN, pNN5% and RMSSD) were reduced in HFD group compared with CTRL one, while HFD Liraglutide prevented the reduction of both indexes induced by high fat diet. For HFD Sitagliptin rats, time-domain indexes did not differ from those of HFD rats and were reduced compared to HFD Liraglutide group, with exception of pNN5% (Fig 2).

Among the groups with a standard diet, CTRL Sitagliptin group showed lower R-R intervals and RMSSD values than CTRL group, while CTRL Liraglutide showed only lower RMSSD values (data not shown).

Additionally, power spectra indexes of HRV (LF, HF and TP) were significantly lower in HFD group compared with control rats. The HFD Liraglutide subgroup presented power spectra indexes higher than HFD one and no significant effects of Sitagliptin treatment (HFD Sitagliptin) were observed, with the exception of TP. The ratio between LF and HF power was higher in HFD compared with CTRL rats. Although incretin administration to rats subjected to high fat diet tended to decrease LF/HF ratio, no significant differences were observed between these animals and HFD rats without treatment. These results clearly suggest that liraglutide is more effective than sitagliptin in preventing autonomic dysfunction induced by high fat diet (Fig 2).

### Cerebral microcirculation

**Fig 3:**
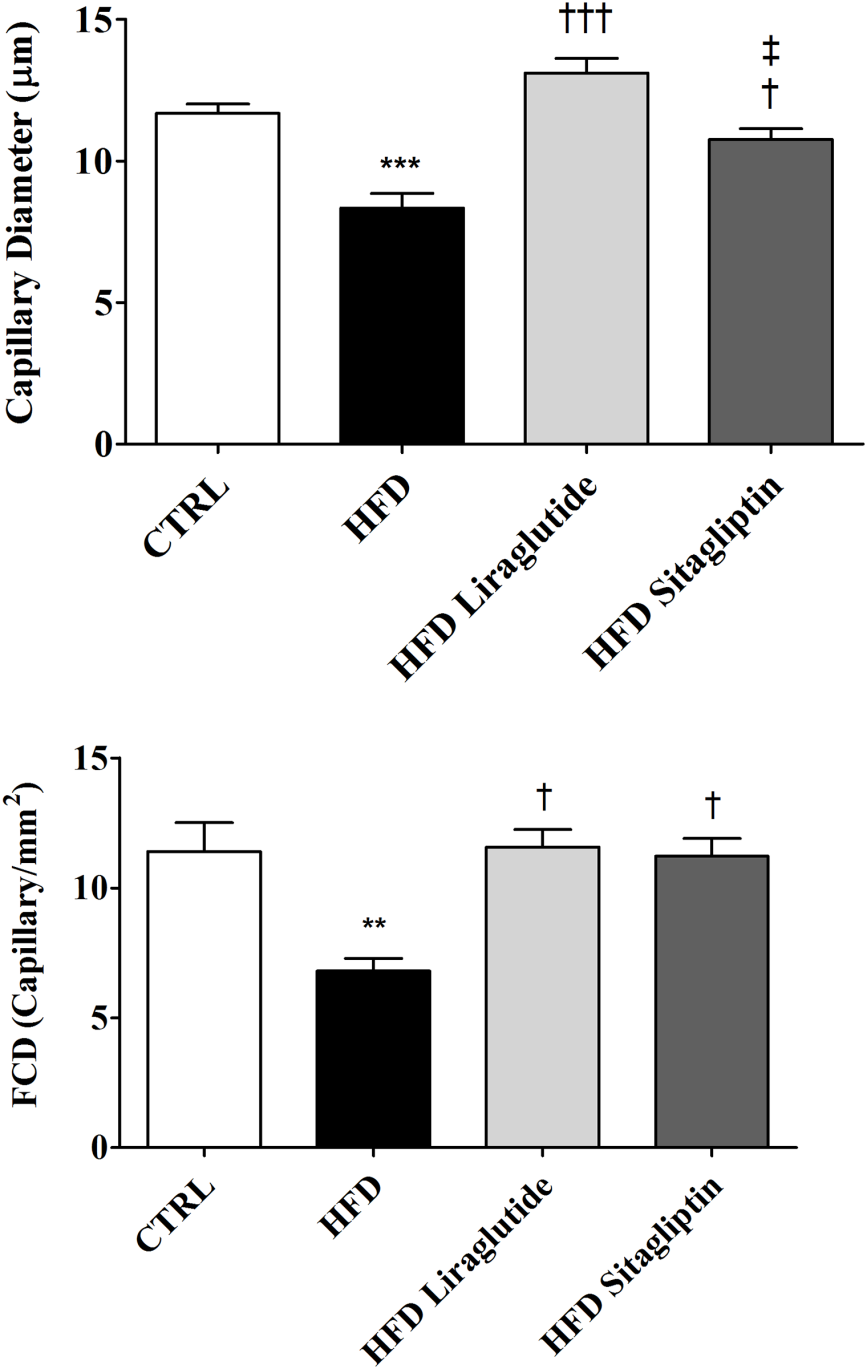
Diameter and functional density of brain capillaries (FCD) after HFD and incretin-based therapies. Data are shown as mean ± SEM; *n* = 5–8. **p<0.01 vs. CTRL; ***p<0.001 vs. CTRL; ^**†**^*p<*0.05 vs. CTRL; ^**†††**^*p<*0.001 vs. HFD and ^‡^*p<*0.05 vs. HFD Liraglutide.

To evaluate the impact of high fat diet on cerebral microcirculation and the therapeutic potential of incretins, the pial circulation was assessed by intravital microscopy (Fig 3).

As shown, reduced values of capillary diameter were observed in HFD rats compared with CTRL ones, while HFD Liraglutide rats showed normalization of capillary diameter values. Although HFD Sitagliptin showed higher capillary diameter compared to HFD and smaller one than HFD Liraglutide, no significant differences were observed when compared to CTRL rats. In addition, functional capillary density of HFD group showed greater reduction in relationship to CTRL rats, while HFD Liraglutide and HFD Sitagliptin presented similar values compared with CTRL, indicating that incretin-based therapies reversed deleterious effects on cerebral microcirculation induced by high fat diet.

### Mitochondrial swelling

**Fig 4:**
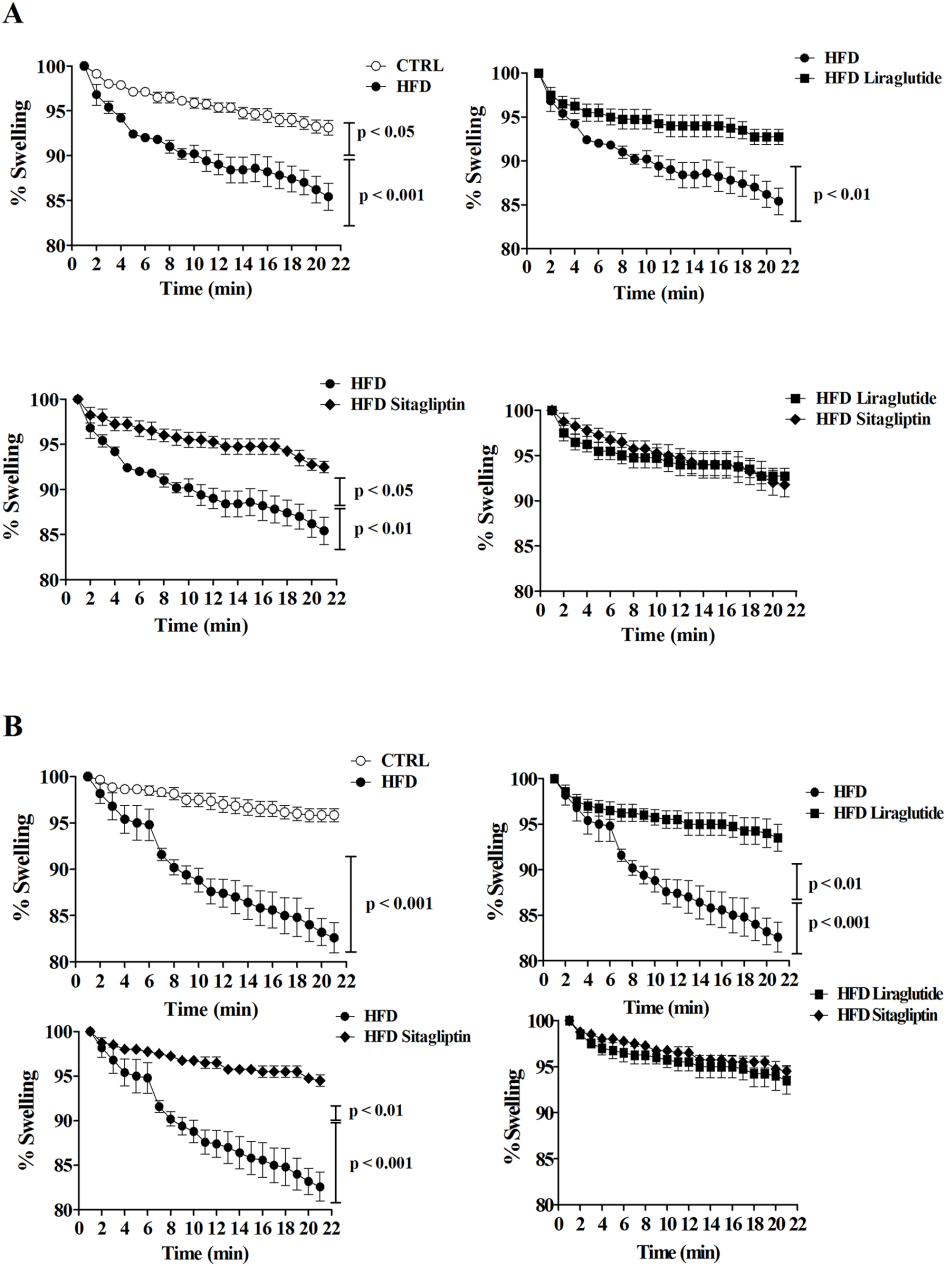
Experimental swelling protocol induced by Ca^2+^. Liraglutide and sitagliptin subgroups showed increased mitochondrial permeability transition pore resistance in brain (a) and heart (b) tissues. Analyses were made in rats having received liraglutide and sitagliptin for 4 weeks after high fat diet during 18 weeks. Data are shown as mean ± SEM; *n* = 5.

To determine the function of mitochondrial permeability transition pores (mPTP) from cerebral cortex and LV, mitochondrial swelling was evaluated in response to Ca^2+^ (Fig 4).

Cortical mitochondria from HFD rats showed greater swelling than CTRL ones. Notably, HFD Liraglutide cortical mitochondria were more resistant to swelling and permeability transition induced by Ca^2+^ than HFD mitochondria, with similar levels of CTRL mitochondria. In addition, mitochondrial swelling of HFD Sitagliptin was greater than HFD rats and similar to CTRL and HFD Liraglutide ones, suggesting that brain mitochondrial integrity was maintained after incretin-based therapies (Fig 4a).

On the other hand, as demonstrated on Fig 4b, LV mitochondria from HFD rats showed greater swelling compared with CTRL rats. Both Liraglutide and Sitagliptin treatments prevented high fat diet-mediated effects, since mitochondrial swelling observed on HFD Liraglutide and HFD Sitagliptin subgroups was significantly smaller than observed on HFD LV mitochondria. These results suggest improved resistance and maintenance of function of mPTP after incretin-based therapies.

## Discussion

Since recent studies have provided evidence for cardiovascular activation as well as the importance of incretin-based therapies for cardiovascular health conditions, new mechanisms underlying deleterious cardiovascular effects of high fat diet need to be investigated after such therapies.

Our initial findings showed results similar to ones previously described, where GLP-1 analogues exert profound influence on body weight reduction, while DPP-4 inhibitor had neutral effects [10–12]. Regarding glycaemia, our results show that the GLP-1 agonist induced better control of autonomic nervous system and glycaemic levels than the DPP-4 inhibitor. The different mechanisms of action of GLP-1 analogues and DDP-4 inhibitors may explain our results.

In general, endogenous GLP-1 has two main routes of action: (1) the most common one, triggering glucose-induced insulin secretion from pancreatic beta-cell and (2) binding to GLP-1R located on afferent neurons within the intestine and portal vein, regulating the firing rate of the vagus nerve and its sending to the brain stem followed by transmission of the enteric signals acting in the gut-brain axis by the hypothalamus [13–14]. In parallel, the most common route of DPP-4 inhibitors is to enhance the hepatoportal concentration of GLP-1, favoring the activation of the gut-brain axis [12]. It is known that during the progress of diabetes and obesity through high fat diet in experimental rodent models, the development of autonomic neuropathy occurs, which could alter the gut-brain axis and the enteric GLP-1 signal, consequently hampering the therapeutic efficacy of DPP4 inhibitors [12].

In addition, an experimental rodent model subjected to high fat diet shows impairment in neurovascular coupling, cerebrovascular dysfunction and worsened short-term outcomes after cerebral ischemia [15]. As reported previously by PET studies exploring human satiation and hypothalamus, GLP-1 has greater influence on cerebral blood flow due to postprandial raise in plasma with GLP-1 being correlated to an increased regional cerebral blood flow [16]. Our intravital microscopy study showed that both, GLP-1 agonist and DPP-4 inhibitor, prevented high fat diet-induced cerebral microcirculation deleterious effects. The mechanisms by which high fat diet cause cerebral microcirculation disturbances have not yet been clearly established, but a pioneer study previously published has suggested that altered cellular metabolism could allow changes in cerebral blood flow and cerebrovascular resistance [17].

As diabetes and obesity are associated to higher production of mitochondrial reactive oxygen species (ROS), leading to mitochondrial and cellular oxidative damage, the major effects of ROS is on opening of mPTP, which contributes to mitochondrial swelling and lysis of cells [18]. A major contribution of this study is that both treatments, GLP-1 analogue and DPP-4 inhibitor, increase brain resistance to mitochondrial swelling protocol as well as their maintenance of capillary brain diameter and functional capillary density.

Although heart weight and cardiac hypertrophy are augmented after long-term feeding with high fat diet, previous studies have reported that incretin-based therapies could reverse these parameters only when feeding normal chow diet. Furthermore, several studies in rodents have shown functional changes in the heart induced by high fat feeding, including increased blood pressure and reductions of contractility (dP/dt^+^) and relaxation (dP/dt^-^) indexes [19]. In addition, incretin-based therapies (GLP-1 analogues and DPP-4 inhibitors) often produce beneficial effects in models with ventricular dysfunction. The underlying mechanisms of incretins are not well defined, but it may involve AMPK since administration of AMPK antagonist (compound C) reversed the beneficial effects of incretin-based therapies [20].

It is known that AMPK can regulate many intracellular signals including energy metabolism and mitochondrial function, making mitochondria a major therapeutic target for heart disease treatment. These findings may help us to explain how incretin-based therapies could reverse the deleterious effects on myocardial function induced by high fat feeding, since both GLP-1 analogue and DPP-4 inhibitor prevented mitochondrial permeability transition in cardiac tissue, associated to increased contractility and relaxation myocardial indexes.

The final result of our study using rats and HFD showed that incretin-based therapies could be associated with changes in biometric, metabolic and neuro-cardiovascular dynamic control. Although GLP-1 analog had demonstrated more efficiency on biometric and metabolic disorders than DPP-4 inhibitor, both presented beneficial results on neuro-cardiovascular functions. Brain and heart mitochondria were the main interplay between neuro-cardiovascular functions after incretin-based therapies, since both GLP-1 analog and DPP-4 inhibitor showed improved mitochondrial resistance during the swelling protocol.

Although no significant differences were observed in SBP we observed a significant increase in DBP of HFD group compared to CTRL. It is already known that DBP is directly related to peripheral vascular resistance [21] that, in turn, is correlated with microvascular dysfunction [22]. Metabolic syndrome induced microvascular dysfunction is not tissue specific, occurring in all tissues [23]. Therefore our results suggest that the increase in DBP may be a consequence of changes in peripheral vascular resistance due to microvascular dysfunction, as observed by the reduction in capillary diameter and density in animals subjected to high fat diet.

Additionally, microvascular function is highly dependent of cellular energetic metabolism homeostasis [24, 25] and Mitochondria have an important role in its control and as such mitochondrial dysfunction has a direct deleterious impact on different tissues [26–30]. At the present study, heart and brain isolated mitochondria of HFD group showed higher Ca_2_^+^ induced experimental stress than the CTRL group, leading to cerebral microvascular dysfunction and hemodynamic unbalance. Liraglutide and sitagliptin treatments are able to normalize these effects, restoring microvascular and mitochondrial function.

Our work was conducted in a perspective of integrative physiology, and therefore mechanisms related to the deleterious effects on the heart and brain function, such as protein expression investigation and mitochondrial function analysis (high-resolution oximetry and mitochondrial membrane potential), are not shown here.

In conclusion, our results present a new mechanism for incretin-based therapies showing that they could act on deleterious cardiovascular effects induced by high fat feeding and may have important contributions on the interplay between neuro-cardiovascular dynamic controls through mitochondrial dysfunction associated to metabolic disorders.
